# PanglaoDB: a web server for exploration of mouse and human single-cell RNA sequencing data

**DOI:** 10.1093/database/baz046

**Published:** 2019-04-05

**Authors:** Oscar Franzén, Li-Ming Gan, Johan L M Björkegren

**Affiliations:** 1 Integrated Cardio Metabolic Centre (ICMC), Department of Medicine, Karolinska Institutet, Novum SE Huddinge, Sweden; 2 Cardiovascular, Renal and Metabolism Translational Medicines Unit, Early Clinical Development, IMED Biotech Unit, AstraZeneca, Pepparedsleden, Mölndal, Sweden; 3 Icahn Institute for Genomics and Multiscale Biology, Department of Genetics and Genomic Sciences, Icahn School of Medicine at Mount Sinai, One Gustave L. Levy Place, New York, NY, USA

## Abstract

Single-cell RNA sequencing is an increasingly used method to measure gene expression at the single cell level and build cell-type atlases of tissues. Hundreds of single-cell sequencing datasets have already been published. However, studies are frequently deposited as raw data, a format difficult to access for biological researchers due to the need for data processing using complex computational pipelines. We have implemented an online database, PanglaoDB, accessible through a user-friendly interface that can be used to explore published mouse and human single cell RNA sequencing studies. PanglaoDB contains pre-processed and pre-computed analyses from more than 1054 single-cell experiments covering most major single cell platforms and protocols, based on more than 4 million cells from a wide range of tissues and organs. The online interface allows users to query and explore cell types, genetic pathways and regulatory networks. In addition, we have established a community-curated cell-type marker compendium, containing more than 6000 gene-cell-type associations, as a resource for automatic annotation of cell types.

## Introduction

Single-cell RNA sequencing (scRNA-seq) is a technology that measures gene expression at the single-cell level (1). scRNA-seq achieves an unprecedented level of resolution and it is increasingly used to examine the cellular architecture of tissues, organs and whole organisms. In contrast to bulk RNA-seq, where gene expression is measured and averaged across thousands of cells, scRNA-seq provides much more detailed information and has generated new insights into cellular states, trajectories and heterogeneities. In a typical scRNA-seq experiment, cells from tissue biopsies are dissociated, RNA is converted to cDNA and libraries are generated containing thousands of transcriptomes. Each transcriptome is tagged using a unique oligonucleotide barcode. Certain sequencing protocols incorporate Unique Molecular Identifiers (UMIs) (2) in their workflows so that PCR duplicates can be removed at the data analysis stage. Compared with bulk RNA-seq, scRNA-seq data contain many zero measurements, caused by dropout events and a cleaner biological signal (3). Several protocols and platforms have been developed for scRNA-seq, for example Drop-seq (4), 10X Chromium and SMART-seq2 (5).

The rapid rise of scRNA-seq has led to the accumulation of massive amounts of sequencing data in public archives [such as the National Center for Biotechnology Information (NCBI) Sequence Read Archive (SRA)], since most journals and funders require that upon publication, sequencing data are released to the public domain. However, deposited data often remain difficult to access as it requires significant pre-processing to become useful for regular analysis. Moreover, while the NCBI SRA is an excellent resource for data storage, there is little to no mechanism for quality control, data curation and annotation. Quick access to published datasets allows researchers to answer new questions using old data, prevents duplication of previous efforts and perhaps most importantly, enables comparisons with in-house data to validate or generate new biological hypotheses. Altogether, there is a strong need from the scientific community for efforts involving collection, curation and integration of scRNA-seq data with bioinformatic workflows into platforms that are easily accessible.

Previous efforts to develop integrative databases for scRNA-seq analysis include scRNASeqDB (6) and SCPortalen (7), the former being limited to 36 pre-processed datasets collected from the Gene Expression Omnibus (GEO) (8). SCPortalen appears relatively limited in scope and does not provide advanced visualization tools since it is more focused on the technical properties of scRNA-seq data. None of the databases provide pre-computed bioinformatic analyses and advanced visualization from a user perspective.

Here, we have developed PanglaoDB—a protocol-agnostic platform for the exploration of scRNA-seq data through a web-based interface. We have collected data and metadata from hundreds of human and mouse studies and processed these data through a unified computational pipeline. In addition to enabling exploration of scRNA-seq experiments, our database provides a manually curated list of cell-type markers that can be incorporated into novel algorithms for inference of cell types. The aim of our work is to provide a frequently updated online single-cell resource to facilitate investigation and hypothesis-free exploration of scRNA-seq data generated by independent academic labs around the world. PanglaoDB unlocks access to more than 1000 single cell experiments, and as such represents the most up to date public resource of curated scRNA-seq data ready for open use by the scientific community.

## Materials and methods

### Web server and interface

The database is hosted on a Virtual Private Server running Ubuntu Linux with four virtual CPUs, 16 GB RAM and 500 GB hard drive space. We decided to use Nginx as web server because it is relatively lightweight and memory-lean. Nginx was configured to use an SSL certificate from Let’s Encrypt. MySQL was used to keep track of data processing steps and leverage the database through the web interface. The interactive view was built using the D3.js JavaScript library and Python scripts for pulling data.

### Data collection and bioinformatics pipeline

Experimental metadata from high-throughput sequencing studies were downloaded from the NCBI SRA (9) (ftp-trace.ncbi.nlm.nih.gov/sra/reports/Metadata/). We used only submissions fulfilling the following criteria: (i) listed without controlled access; (ii) classified as transcriptomic; and (iii) species is human (TaxID = 9606) or mouse (TaxID = 10090). We then searched abstracts, titles and sample identifiers using the following, case insensitive, regular expression: /(single cell seq|drop\-^*^seq|scrna|single cell rna-seq|10x\s^*^(genomics|chromium)|smart-seq2)/. The sequence data were then examined to determine if barcodes and/or UMIs were encoded in the submission; submissions without proper barcode information were discarded from further processing. Submissions that passed the filtering criteria were manually inspected to make sure that these were true scRNA-seq studies. In SRA terminology, each submission receives a unique identifier (/SRA[0-9]+/). A submission may consist of more than one sample (/SRS[0-9]+/), which may consist of more than one sequencing run (/SRR[0-9]+/). In PanglaoDB, a sample is a dataset consisting of gene expression measurements from cells originating from a common biological source or experiment. Multiple runs for each sample were merged into one file. Some studies prefer to deposit each cell with their own SRS accession, in those cases the sample is referred to by their SRA accession. We used the prefetch program in the sratoolkit v.2.9.0 to streamline downloading of the sequencing data. Submissions to the ERA and DRA databanks were not included due to incompatibility with prefetch.

Sequencing reads were extracted from SRA files using either fastq-dump or vdb-dump. For data generated using 10X Chromium, barcodes and UMIs were assumed to be 16 and 10 bp in length, respectively. For data generated using Drop-seq, barcodes and UMIs were assumed to be 12 and 8 bp in length, respectively. Barcode and UMI sequences were appended to the read identifier, which is the first line in a FASTQ record: @[readid]_[barcode]_[UMI]. SAM to BAM conversion, sorting and indexing were performed with samtools (10) v.1.8 or sambamba (11) v.0.6.7. The featureCounts program of subread (12) v.1.6.2 was used to add gene information to the BAM file. If the single-cell protocol used UMIs, we performed UMI deduplication using UMI-tools (13) v.0.5.3 (parameters: “--no-sort-output --method unique --gene-tag=XT --per-gene –per-cell”).

All sequencing reads were mapped with hisat (14) v.2.1.0, which was selected because of its low memory footprint; only reads with mapping quality ≥60 were retained. Mouse and human reads were mapped to GRCm38 and GRCh38, respectively. GENCODE (15) v.27 was used as genome annotation (exons of the same gene were collapsed into ‘meta genes’; gene identifiers were set to [gene symbol]_[ENSEMBL ID]).

Counting of reads was performed with the UMI-tools count command or a custom Python script. Raw read counts were converted into a sparse R matrix where columns represented cells and rows represented genes.

The data processing pipeline performs basic quality control; for the most part we assume the data are of good quality since it has been released to a public archive. We only included cells with at least 1000 uniquely mapped reads after UMI deduplication. Only clusters with at least 10 cells are used in analyses.

### Clustering and cell-type inference

Read counts per cell were adjusted for total number of reads and then log_2_(*x* + 1) transformed (*x* is the expression value for one gene in one cell, either as scaled counts or RPKM). A pseudo-count of 1 was added to all RPKM values prior to log_2_ transformation to avoid log_2_(0) = −Inf for genes with zero expression [studies using RNA-seq often do this, for example (16)]. Cell doublets were predicted with the Scrublet software (https://github.com/AllonKleinLab/scrublet) and the top 5% cells with the highest scores were removed. Cell clustering was performed with the FindClusters function in Seurat (17) v.2.3.2 using the PCA method (parameters: dims.use = 1:75, resolution = 0.8, k.param = 10). The resolution parameter was manually evaluated, and we decided to proceed with 0.8 to generate a smaller number of large clusters. Samples were clustered separately. Dimensionality reduction for visualization was performed using t-distributed stochastic neighbor embedding (t-SNE) (18) and uniform manifold approximation and projection (UMAP) (19).

The database of cell type markers was compiled by manual curation of thousands of published articles and abstracts, and by querying internet search engines with strings such as ‘GENE1 is expressed in ^*^ cells’. We did not require that gene markers had to be specific for a cell type, since our approach borrows information from multiple markers rather than relying on a single marker. For some cell types, when canonical markers were unambigious, we extended the list of putative cell-type markers by examining expressed genes in the particular single-cell cluster. Hence, our marker compendium is a mix of canonical and novel markers.

To determine the cell type of a cell cluster, we perform the following steps: for every cell cluster *k* in a sample *s*, we iterate over all genes $i=1\dots N$ and calculate the median expression per gene over normalized gene expression measurements so that gene expression for a cell cluster is represented by a vector ${v}_{s,k,i}$. The cell-type identity is then determined using the collection of cell-type marker genes. Some marker genes are found in multiple cell types and are less informative for cell-type inference. A simple workaround for such ‘multi cell type’ markers would be to remove them. However, removing markers would decrease sensitivity and inflate type II errors. We decided to use down-weighing of genes based on their frequency, an idea borrowed from gene set analysis (20), which relates the weight $w(g)$ for a gene to its frequency across gene sets (cell types in our case), $f(g)$, using a monotonically decreasing function:

$$ w(g)=1+\sqrt{\frac{\mathit{\max}(f)-f(g)}{\mathit{\max}(f)-\mathit{\min}(f)}}. $$

Thus, weights are bounded between 1 and 2, where genes occurring in many cell types receive weights closer to 1 and more specific genes will get doubled weights ($w=2$). Next, to define the putative cell type, we calculated a cell-type activity (CTA) score, similar to (21), for all of the >150 possible cell types. The CTA score estimates the ‘activity’ of marker genes, down-weighing the contribution of broad markers and adjusting the score for the total number of markers for the cell type:

$$ {S}_{j,k}=\left({\sum}_{i=1}^N{Z}_{k,j,i}\bullet {w}_i\right)\big/\sqrt[3]{N}. $$

*S_j,k_* is the CTA score for cell-type *j* in cell cluster *k* and *N* is the total number of marker genes. *Z* is normalized gene expression counts. For a given cell cluster, CTA scores are then ranked from highest to lowest and the top-ranking cell type is selected as the ‘winner’. A *P* value is computed using a one-sided Fisher’s exact test (hypergeometric test) on genes being expressed and not. Genes were defined as expressed if expression was >0. A false discovery rate was calculated with the Benjamini–Hochberg procedure (22); if the adjusted *P* value is >0.05 for the top-ranking cell type, it is set to ‘Unknown’.

We validated our method using a subset of included samples where the reported cell type was known. The majority of included samples represented whole tissues, in which case there is more than one cell type. However, some single cell studies have targeted one specific cell type by flow cytometric sorting of cells using their surface markers. In such cases it is possible to compare if the predicted cell type matches the ‘biological’ cell type. We identified 17 independent datasets with homogeneous cell-type populations, where the biological cell type has been reported (this information is available as metadata in the NCBI SRA; Supplementary Table 1). In all 17 cases the biological cell type was identical to the cell type predicted by our method. As a side note, in one sample (SRS2781556), the biological annotation was microglia, whereas our prediction found that the bulk of cells was microglia and a small cell cluster was neutrophils. The most likely explanation to this relates to that microglia and neutrophils share the same myeloid origin, thus the isolation protocol have captured a few neutrophils. We embarked on an orthogonal validation approach, based on tissue samples and the expectation to find a certain cell type. For example, a tissue sample from liver is expected to contain hepatocytes (the most abundant cell type in liver) and a tissue sample from the central nervous system can be expected to contain glia cells. We randomly selected 13 tissue samples from a wide range of tissues and examined if the ‘prominent’ or ‘expected’ cell type of the particular tissue matched one of the types predicted by our method (Supplementary Table 1). In all 13 examined datasets, there is an overwhelming consistency between the expected cell type and the predicted.

**Figure 1:**
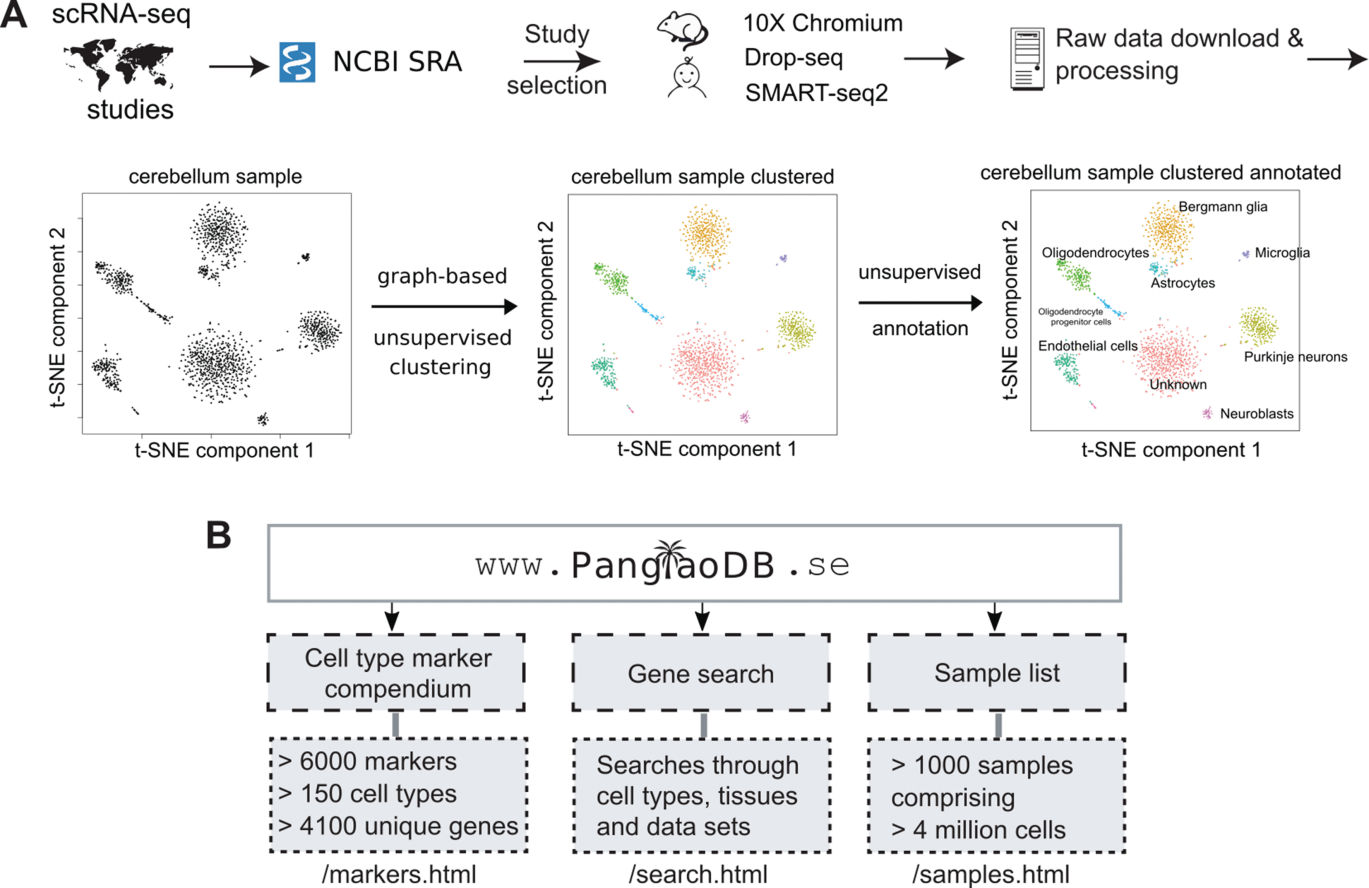
Overview of the database and features. (**A**) Data from selected single cell RNA sequencing experiments were downloaded from NCBI SRA and filtered according to a number of different criteria. Only mouse and human data were included. The bulk of the data came from three scRNA-seq platforms/protocols (10X Chromium, Drop-seq and SMART-seq2). Data were processed in a standardized bioinformatics pipeline. The example shows the sample SRS3059959 from mouse cerebellum. Analyses were conducted on the final processed data, and files were uploaded to PanglaoDB. (**B**) The three entry points of PanglaoDB are (i) the cell marker compendium, (ii) the gene search function and (iii) the sample list.

### Gene search

We experimented with several metrics to represent the expression of a gene within one cell cluster (median, geometric mean, arithmetic mean and harmonic mean), and we eventually settled with the median being the most useful in most situations. To allow comparison of expression levels between different datasets, we computed gene expression ranks; i.e. the most highly expressed gene of a cell cluster always has rank 1.

### Regulons

Regulons were identified by largely following what was described in (23) and (24). First, we derived a co-expression network for each cell cluster using stochastic gradient boosting (GRNBoost2: https://arboreto.readthedocs.io/en/latest/) (25). We also explored creating co-expression networks using GENIE3 (26) and WGCNA (27), the former was deemed too computationally expensive and the latter did not work well due to the sparse properties of the single cell sequencing data. Co-expression networks were centered on transcription factors; i.e. the central node in each subnetwork was a transcription factor. We used a list of curated transcription factors from TFCat (28). We collected an extensive set of 4104 positional weight matrices (PWMs) from various online sources [JASPAR (29), HOCOMOCO (30), SwissRegulon (31), UniPROBE (32) and CIS-BP (33)], representing experimentally derived transcription factor binding site motifs of 560 transcription factors (the collection has multiple and overlapping motifs for several transcription factors).

We used FANTOM5 CAGE peaks to compile a list of transcriptional start sites (34). For genes without a CAGE peak we simply used the most 5′ UTR of the longest isoform. A window of +/− 10 kb around each transcriptional start site of all protein-coding and long non-coding RNA genes were extracted and searched using the PWMs. The program fimo (part of the meme v.4.12.0 package) (35) was used for searching both DNA strands (--max-strand parameter). The choice of fimo was based on the conclusions from (36). Previous studies have found that transcription factor binding motifs are often clustered into cis-regulatory modules (37). Fimo gives multiple hits for each gene (20 kb sequence), likely representing redundant motifs. We rescored the fimo output by summing over the score for each motif-gene pair, and then ranking the genes according to this new score.

An enrichment test was performed to examine if co-expressed genes were enriched in the top-ranking genes for a motif (we limited the test to include only the top 200 ranking genes for any motif). For a set of co-expressed genes under the putative control of a certain transcription factor, we calculated the area under the curve for all motifs as a measurement of the genomic background. In the original paper (24), the authors used a Z-score to determine the significance of a motif compared to the genomic background. However, we found that in many cases the resulting distribution was not Gaussian. Instead we used a kernel density estimate and integrated over the curve to yield an estimate of the significance (AUC<0.05 was used as significance threshold). The enrichment test was implemented in R using sfsmisc and DescTools as external packages.

### Gene set activity

We used MSigDB (38) v.6.1 as input signatures for gene set activity (GSA) calculations. GSA was calculated as described in (21). To test for enrichment in gene sets we used a one-sided Fisher’s exact test. Bonferroni correction was used to correct for multiple testing (the α parameter was set to 0.01). We only tested gene sets with at least 10 genes and not more than 500 genes. For mouse samples, we restricted the analysis to one-to-one human–mouse orthologs as defined by Ensembl BioMart.

### Cell cycle analysis, differential expression and disease associations

Cell cycle analysis was conducted using the cyclone function in the scran R package (39). For every cell cluster, each cell was assigned into G1, G2M and S phases. Differential expression analysis was implemented based on the FindMarkers function of the Seurat package (17). Disease associations for genes were extracted from the eDGAR database (40).

## Results and discussion

### Design and general description of the database

The aim of PanglaoDB is to unlock access to scRNA-seq data through a simple and user-friendly interface that allows analysis, visualization and biological interpretation of gene expression data from multiple tissues and studies. Figure 1A shows the data flow from the NCBI SRA (9). Most submissions to the NCBI SRA have a corresponding submission to the GEO (8). We decided not to use the GEO submission because it is currently difficult to reconcile datasets from GEO because of inconsistencies in data formats and genome annotations. Furthermore, we reasoned that remapping and recounting sequencing reads from scratch have the advantage of homogenizing the analytical procedures in terms of algorithms, reference genomes and annotations. Raw sequencing data were identified and downloaded from the NCBI SRA; data were verified to be true scRNA-seq data by manual inspection. Sequencing reads were processed in a standardized bioinformatics pipeline, involving mapping to reference genomes and basic quality control. Cells in each sample were clustered and cell types were inferred (Figure 1A). The three main entry points to the database are the sample list, the search function and the marker compendium (Figure 1B). Currently, we have integrated 845 and 209 single-cell samples from mouse and human, respectively. Altogether, these samples contain data from more than 4 million cells. We anticipate these numbers to grow substantially over the next few years as the use of scRNA-seq continues to be adopted across the biological and medical disciplines.

**Figure 2:**
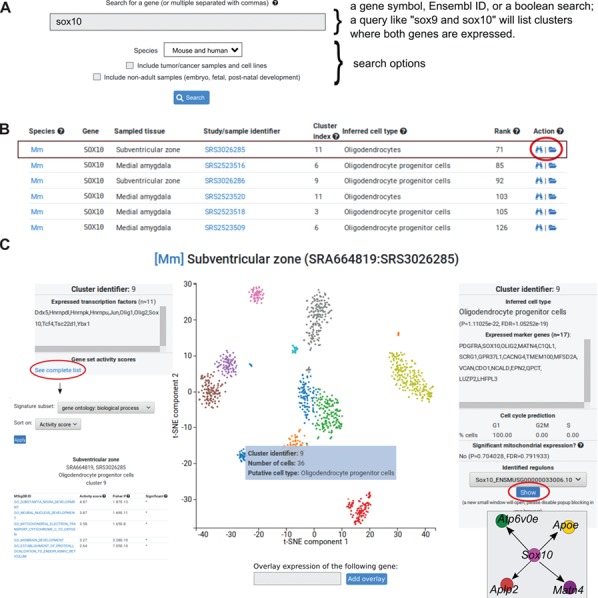
Data analysis visualization interface. (**A**) The search window box is one of the primary entry points to the data. Genes are queried using their gene symbols. The search function also recognizes any non-ambiguous gene aliases. Multiple genes can be separated by commas. (**B**) Partial search results from a query for *Sox10* (first six rows shown). Each row represents one cell cluster where the gene is expressed. Columns correspond to the following: species (Mm = mouse, Hs = human), gene symbol, sampled tissue, study/sample identifier, cluster index (each sample is clustered and clusters are identified by their corresponding 0-indexed identifier), the inferred cell type of the cluster, gene expression is shown as ranks, and actions. The folder icon (indicated with a red circle) will open a more detailed view of the dataset where the cell cluster is located. (**C**) The interactive view, showing the 2D projection from t-SNE of one dataset (SRS3026285) from the subventricular zone. Colors correspond to clusters. Hovering the mouse over a cluster will open a transient window (blue box) with three rows: cluster identifier, number of cells in the cluster and putative cell type. When clicking on the cluster, the left and right boxes will open. The left box shows the number of expressed transcription factors in the selected cluster (the example lists 11 transcription factors in cluster 9). To explore gene set activities, the blue link can be clicked and a separate window will open. The right boxes shows the inferred cell type of the cluster (in the example, Oligodendrocyte progenitor cells), a *P* value from a hypergeometric test and a computed false discovery rate. Expressed marker genes are indicated in the box. Below boxes indicate number of cells in three phases of the cell cycle. The final box can be used to explore regulons.

All included samples can be browsed by navigating to the ‘samples’ entry point; the list can be filtered by species, protocol and/or sorted on several different attributes (time added, tissue, protocol and number of cells). To explore a particular sample of interest, the ‘samples’ entry point should be used; a link is available to the right, ‘view’, which opens a new page containing metadata, summary statistics and 2D projections of the gene expression data. While t-SNE is one of the most used algorithms to project scRNA-seq data to a low-dimensional space, PanglaoDB also offers projections generated using UMAP. Examples of metadata that are shown for each sample are species (mouse or human), tumor/cancer sample status, if it is a primary adult tissue sample, the employed scRNA-seq protocol, sequencing instrument and sampled tissue/organ. Summary statistics include number of cells, number of expressed genes, median number of expressed genes per cell and number of clusters. To show gene expression for a specific gene in the selected dataset, one or multiple (comma separated) gene symbols can be entered in the search box with the label ‘Gene search’, which will open a bar plot where each bar corresponds to a cell and colors correspond to clusters. When querying individual samples, read counts have been scaled to library size and log_2_(x+1) transformed (full-length mRNA protocols also adjust for mRNA length). To perform detailed exploration of gene expression, two buttons containing the word ‘interactive’ are available below the 2D projection plot. The interactive view can be used to explore gene set signatures, cell cycle states, transcription factors and other properties. PanglaoDB incorporates regulon predictions for all mouse datasets. Regulon data are accessible from the interactive projection (shown by clicking ‘view’ on the right side in the sample list); clicking on a cell cluster will open a panel to the right, showing predicted regulons for the cell cluster. Differential expression analysis can be performed between different cell clusters of the same sample from the interactive view.

Each dataset in PanglaoDB represents one biological sample and datasets can be uniquely identified using its corresponding SRS accession. Raw read counts can be downloaded as an R sparse matrix object and as a compressed plain text file, so that researchers are not limited by pre-computed analyses. If the scRNA-seq protocol is based on full-length mRNA sequencing, count data are also available as RPKM values. Due to disk space limitation, we don’t store sequencing reads or alignments after processing and analyses are completed; the original reads can nevertheless be downloaded from the NCBI SRA if they are needed by the user. Bulk download of all datasets in PanglaoDB can be performed through the main menu (an archive file in tar format, current file size is ~22 GB).

### Gene expression markers for cell-type inference

Useful biological insights from scRNA-seq data rely on accurate inference of cell-type identity. At the time when this work was initiated, there was no published database or comprehensive list of genes that can be used for automatic cell-type assignment [CellMarker (41) came out when this manuscript was in preparation]. We therefore compiled a compendium of 6631 gene markers mapping to 155 cell types. The compendium was created using manual examination of the literature. We distinguished markers specific to mouse and human. It is possible for a gene marker to map to more than one cell type. Cell types were subsequently grouped into organs (*n* = 26) and germ layers (*n* = 3). The typical cell type has 28 (median) gene markers assigned, but certain broad cell types such as fibroblasts have more than 100 assigned markers.

Assignment of genes to cell types requires broad expertise; we implemented a community-based approach to curation of gene expression markers. Any gene marker can be flagged by clicking a ‘flag’ link, which will mark it for review; a mechanism to propose new markers is also available. For each gene, the marker database computes an ubiquitousness index (UI), which is an indicator of how often the gene is expressed in cell clusters. UI takes values between 0 and 1. Values toward 1 indicate the gene is expressed in more cell clusters, indicating the gene to be involved in housekeeping tasks. Sensitivity is also calculated for every marker-cell type, representing a measure of how frequently the marker identifies the cell type uniquely.

The list of cell type markers consists of the following columns: (i) species, (ii) gene symbol, (iii) UI, (iv) sensitivity, (v) marker count (number of cell types this marker is used in; only shown if browsing a specific marker), (vi) cell type, (vii) germ layer, (viii) organ, (ix) gene aliases, (x) product description and (xi) if the gene has been associated with any disease (Y for Yes, hovering the mouse over the ‘Y’ shows names of diseases). Canonical markers are shown in green color. The complete list can be downloaded as a tab delimited file for easy loading into scripts (link ‘get tsv file’).

### Search gene expression across datasets, cell types and studies

PanglaoDB has a central search function that allows users to search through the entire collection of included datasets (Figure 2). Each row listed by a search query represents one cell cluster. The search function finds cell clusters where the median expression of the queried gene is higher than 0. To allow comparison of expression levels across studies, we provide the gene expression rank within the cell cluster. Users can type one or more comma-separated gene symbols or Ensembl identifiers. Common aliases of most genes can also be used. Executing a search query such as one for *Sox10* will list cell clusters (mouse and/or human) where the median expression is higher than 0. The user can choose to include tumor/cancer samples in the search as well as non-adult samples, which represent experiments on developmental phases (embryo, fetal and post-natal periods). The search box supports the boolean operators ‘AND’ and ‘NOT', which can be used to list cell clusters where certain genes are expressed and not expressed.

The columns in the search output correspond to (i) a two-letter abbreviation of the species (Mm=*Mus musculus*, Hs=*Homo sapiens*); (ii) gene symbol; (iii) the tissue or body site where the sample originates from; (iv) the SRA sample accession; (v) the cluster index (follows the Seurat naming convention for cell clusters); (vi) the inferred cell type (based on the marker compendium); (vii) the expression rank of the gene within the cell cluster; and (viii) action links where the rightmost link (folder icon) leads to the t-SNE plot for the sample. In addition, each search query is summarized by a bar plot showing the number of cell clusters grouped by cell type (Y-axis is number of cell clusters and X-axis is distinct cell types found). Hovering the mouse over the bars will show a brief description of the cell type.

## Conclusions and future perspectives

We have developed an easy to use scRNA-seq platform, PanglaoDB, enabling researchers to explore scRNA-seq data from mouse and human using an interactive interface. We have compiled a comprehensive compendium of more than 6000 gene-cell markers, which we use to annotate cell clusters of samples included in the database. In addition, the compiled list of markers can be used in development of novel algorithms for cell type inference. PanglaoDB will be actively maintained and developed to accommodate the needs of the biomedical research community. Further development of the platform is planned to involve features to submit custom data and cell lineage mapping. In conclusion, PanglaoDB is to our knowledge the most easy to use, well-purposed scRNA-seq data assembly to date providing an interface to published scRNA-seq data.

## Supplementary Material

Supplementary_Table_1_baz046

## Notes

**Database URL:** https://panglaodb.se/
